# Liraglutide Enhances Cell Viability and Reduces Oxidative Stress in Hyperglycemic H9c2 Cardiomyocytes

**DOI:** 10.3390/medicina61101754

**Published:** 2025-09-26

**Authors:** Sinem Durmus, Zeki Dogan, Dilek Duzgun Ergun, Mahmut Ozdemir, Hakan Sahin, Gozde Erkanli Senturk, Remise Gelisgen, Hafize Uzun

**Affiliations:** 1 Department of Medical Biochemistry, Faculty of Medicine, İzmir Kâtip Celebi University, Izmir 35620, Türkiye; 2 Department of Cardiology, Faculty of Medicine, Istanbul Atlas University, Istanbul 34403, Türkiye; drzeki@yahoo.com; 3 Department of Biophysics, Faculty of Medicine, Istanbul Aydin University, Istanbul 34295, Türkiye; dilekergun@aydin.edu.tr; 4 Department of Cardiology, Faculty of Medicine, Istanbul Beykent University, Istanbul 34475, Türkiye; mahmutozdemir@beykent.edu.tr; 5 Department of Histology and Embryology, Cerrahpasa Faculty of Medicine, Istanbul University—Cerrahpasa, Istanbul 34098, Türkiye; hakan.ahin@gmail.com (H.S.); gozde.erkanlisenturk@iuc.edu.tr (G.E.S.); 6 Department of Medical Biochemistry, Cerrahpasa Faculty of Medicine, Istanbul University—Cerrahpasa, Istanbul 34098, Türkiye; remise.gelisgen@iuc.edu.tr; 7 Department of Medical Biochemistry, Faculty of Medicine, Istanbul Atlas University, Istanbul 34403, Türkiye; huzun59@hotmail.com

**Keywords:** cardiomyopathies, diabetes mellitus, type 2, hypoxia-inducible factor 1, alpha subunit, liraglutide, oxidative stress

## Abstract

*Background and Objectives*: Cardiovascular disease remains a leading cause of mortality in Diabetes mellitus (DM), where chronic hyperglycemia induces oxidative stress, mitochondrial dysfunction, and hypoxia in cardiomyocytes. Liraglutide (Lir), a glucagon-like peptide-1 receptor agonist, is widely used for type 2 DM management and has been shown to exert cardioprotective and antioxidant effects. This study aimed to evaluate whether Lir mitigates hyperglycemia-induced oxidative and hypoxic stress in H9c2 cardiomyoblasts while preserving cellular ultrastructure. *Materials and Methods*: H9c2 cells were cultured under normoglycemic (5.5 mM) or hyperglycemic (30 mM) conditions, with or without Lir. Cell viability was assessed using MTT assay. Ultrastructural changes were examined by transmission electron microscopy (TEM). Hypoxia-inducible factor-1α (HIF-1α), lipid peroxidation markers (LOOH, MDA), advanced oxidation protein products (AOPP), and total antioxidant capacity (TAC) were quantified by spectrophotometric assays. *Results*: MTT assays revealed that Lir significantly improved cell viability under hyperglycemic conditions and the EC_50_ was 1.05 ± 0.06 μM after 48 h of treatment. Under HG, HIF-1α, lipid hydroperoxides (LOOH), malondialdehyde (MDA) and advanced oxidation protein products (AOPP) increased and total antioxidant capacity (TAC) decreased (*p* < 0.001, for all); Lir significantly reversed these changes, restoring values to near-NG levels. Ultrastructural analysis of HG + Lir-treated cells revealed reduced granules, increased vacuolization, and slight rough endoplasmic reticulum dilatation, though mitochondria appeared normal. *Conclusions*: Lir significantly attenuated oxidative stress and cellular injury in cardiomyocytes under hyperglycemic conditions, improving viability, modulating HIF-1α expression, and restoring antioxidant balance. These findings support a dual role for Lir in diabetic cardiomyopathy: glucose-independent cytoprotection and regulation of mitochondrial and hypoxia pathways, highlighting its therapeutic potential beyond glycemic control.

## 1. Introduction

According to the 2025 IDF Diabetes Atlas, one in nine adults (20–79 years) has diabetes, more than 40% of whom are undiagnosed. This is projected to increase to one in eight adults (~853 million) by 2050. Cardiovascular diseases are one of the leading causes of morbidity and mortality in Diabetes Mellitus (DM) [1]. Chronic hyperglycemia plays a role in diabetic cardiomyopathy by causing oxidative stress, inflammation, and mitochondrial dysfunction in cardiomyocytes [2].

Hyperglycemia disrupts the pro-oxidant/antioxidant balance by increasing the production of reactive oxygen species (ROS) and causes lipid, protein, and DNA damage, leading to apoptosis and organ dysfunction. Oxidative stress can be assessed by biomarkers such as lipid hydroperoxides (LOOH) and malondialdehyde (MDA), which indicate lipid peroxidation, and advanced oxidation protein products (AOPP), which reflect protein oxidation and inflammation. Total antioxidant capacity (TAC) assesses non-enzymatic antioxidant defense [3,4,5]. Hyperglycemia also affects hypoxia-inducible factor-1 alpha (HIF-1α) activity and is associated with metabolic disturbance and mitochondrial distress in cardiomyocytes [6].

Glucagon-like peptide-1 (GLP-1) receptor agonists and sodium-glucose cotransporter 2 (SGLT2) inhibitors have been shown to provide significant cardiovascular protection in patients with type 2 DM (T2DM). This benefit is largely attributed to their anti-inflammatory and anti-oxidative effects, which help prevent cardiovascular damage and improve overall cardiac function [7,8].

Liraglutide (Lir), a glucagon-like peptide-1 receptor (GLP-1R) agonist, is widely applied to treat T2DM as it can improve hyperglycemia without any risk of hypoglycemia. Lir imitates endogenous GLP-1 in stimulating insulin secretion by the cAMP/PKA pathway in β-cells and inhibiting the release of glucagon from α-cells. Apart from glycemic management, Lir has cardioprotective, anti-inflammatory, and antioxidant effects since cardiomyocytes also possess GLP-1Rs [9,10,11,12]. Previous studies report that GLP-1R agonists control oxidative stress pathways in the heart and other tissues [13,14,15]; however, an integrated appraisal in cardiomyocytes that concurrently interrogates HIF-1α, lipid and protein peroxidation (LOOH, MDA, AOPP), TAC, and organelle ultrastructure under defined glycemic states remains limited. Here, we hypothesized that Lir would mitigate hyperglycemia-induced oxidative and hypoxic stress in H9c2 cardiomyoblasts and preserve cellular architecture. We tested this by coupling quantitative biochemical readouts with transmission electron microscopy (TEM). The distinct novelty of our study is that it combines these complementary endpoints to delineate a coherent cytoprotective profile for a Lir in cardiomyocytes, thereby refining the mechanistic context for GLP-1R signalling in diabetic cardiomyopathy.

## 2. Materials and Methods

### 2.1. Chemicals

H9c2 (2-1) rat cardiomyocyte cell line, a subclone of the original clonal cell line derived from embryonic BD1X rat heart tissue that exhibits many of the properties of skeletal muscle, was obtained from the American Type Culture Collection (ATCC, Manassas, VA, USA). H9c2 (2-1) [16]. Cells were cultured in complete medium containing 90% Dulbecco’s Modified Eagle’s Medium (DMEM, low glucose, Gibco, Thermo Fisher Scientific, Waltham, MA, USA), 10% heat-inactivated fetal bovine serum (FBS, Euroclone S.p.A., Pero, Milan, Italy), 1% antibiotic solution (100 U/mL penicillin and 100 U/mL streptomycin, Euroclone S.p.A., Pero, Milan, Italy), and 2 mM L-glutamine (Euroclone S.p.A., Pero, Milan, Italy) at 37 °C in a humidified atmosphere with 5% CO_2_. Upon reaching 80–90% confluence, cells were detached using 0.25% Trypsin-EDTA (T4049, Sigma-Aldrich, St. Louis, MO, USA), washed with Dulbecco’s Phosphate-Buffered Saline (DPBS, Euroclone S.p.A., Pero, Milan, Italy), and centrifuged at 1200 rpm for 3 min. The supernatant was discarded, and the pellet resuspended in fresh medium. Cells were seeded into 96-well plates (1 × 10^4^ cells/well) and incubated at 37 °C in 5% CO_2_.

### 2.2. MTT Assay and Cell Viability

Cell viability was assessed using the MTT assay (3-(4,5-dimethylthiazol-2-yl)-2,5-diphenyltetrazolium bromide; Sigma-Aldrich, St. Louis, MO, USA) following exposure of H9c2 cells to various concentrations of Lir. Cells (1 × 10^4^/well) were seeded in 96-well plates and treated with Lir at 0.156–10 µM for 24, 48, and 72 h at 37 °C in 5% CO_2_. Untreated cells served as the control. After incubation, MTT solution (20 µL, 5 mg/mL) and 100 µL fresh medium were added to each well. After 3 h, 100 µL of DMSO (Sigma-Aldrich, St. Louis, MO, USA) was added, and absorbance was read at 570 nm using a Multiskan GO microplate reader (Thermo Fisher Scientific, Waltham, MA, USA). Assays were conducted in triplicate with six replicates per group. EC_50_ values were calculated using GraphPad Prism 9 (GraphPad Software Inc., San Diego, CA, USA) for 24, 48, and 72 h treatments, and experimental groups were established based on the 48 h EC_50_ [16]. All experiments were performed with six independent biological replicates (*n* = 6).

### 2.3. Hyperglycemic Model and Experimental Grouping

A hyperglycemic cardiomyocyte model was established by incubating H9c2 cells in DMEM containing 30 mM glucose for 48 h (HG group) [17,18]. Experimental groups included: normal glucose control (NG; 5.5 mM glucose), hyperglycemic control (HG), Lir-treated (Lir; 1 µM for 48 h), normal glucose + Lir (NG + Lir), and hyperglycemic + Lir (HG + Lir). All experiments were performed with six independent biological replicates (*n* = 6). Following treatment, cell lysates were prepared using 1× RIPA lysis buffer with protease inhibitor cocktail (Merck KGaA, Darmstadt, Germany), as previously described [16] and stored at −80 °C until analysis.

### 2.4. Transmission Electron Microscopic Examination

H9c2 (2-1) cells were seeded into 6-well plates (3 × 10^5^ cells/well), and experimental groups were prepared as previously described [16]. After detachment with 0.25% trypsin-EDTA and PBS washing, cells were fixed in 2.5% glutaraldehyde (4 °C, 1 h), post-fixed with osmium tetroxide, and embedded in 2% agar. Dehydration was followed by treatment with propylene oxide, a propylene oxide-araldite mixture, and pure araldite for resin embedding. Semi-thin sections were cut using an ultramicrotome (Reichert UM3, C Reichert Optische Werke AG, Vienna, Austria), and ultra-thin sections were mounted on copper grids for ultrastructural evaluation by TEM (JEOL JEM-1011, Tokyo, Japan).

### 2.5. Measurement of HIF-1α and Oxidative Stress Biomarkers

HIF-1α levels were quantified in cell lysates using a commercial ELISA kit (Cat. No: E0422Hu, Bioassay Technology Laboratory, Shanghai, China), following the manufacturer’s protocol. The intra- and inter-assay coefficients of variation were <4.9% and <10%, respectively, as reported by the manufacturer.

Lipid peroxidation was evaluated using two markers: LOOH, measured spectrophotometrically via Fe^2+^ oxidation in the presence of xylenol orange, and MDA, determined by a modified TBA method, both as previously described [16]. Protein oxidation was assessed by measuring AOPP, while TAC was evaluated using a modified ferric reducing antioxidant power (FRAP) assay, both as previously described [16]. All assays were optimized for cell lysate samples and adapted to a microplate format.

### 2.6. Statistical Analysis

The distribution of the data was tested using the Shapiro–Wilk test and confirmed to be normal. EC50 values are expressed as mean ± SEM. All other data are presented as mean ± SD, unless otherwise indicated. For comparisons among groups, one-way ANOVA was performed, and F values with degrees of freedom are reported in the Section 3. Tukey’s post hoc test was used for pairwise comparisons. Pearson correlation analysis was applied for correlation studies. Bonferroni correction was used for multiple group comparisons when appropriate. *p*-value less than 0.05 was considered significant. All statistical analyses were performed using JASP 0.19.1 (University of Amsterdam, Amsterdam, The Netherlands).

## 3. Results

### 3.1. Lir Concentrations’ Effects on the Cell Viability of H9c2 Cells

**Figure 1:**
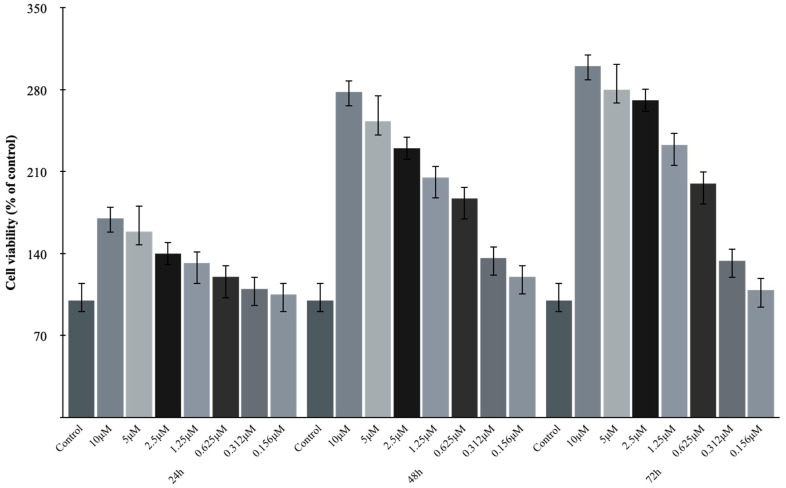
The MTT assay results and effects of indicated concentrations of Lir on the cell viability are presented in the H9c2 cardiomyocyte cells. Data are presented as mean ± SEM. *n* = 6 independent experiments.

**Figure 2:**
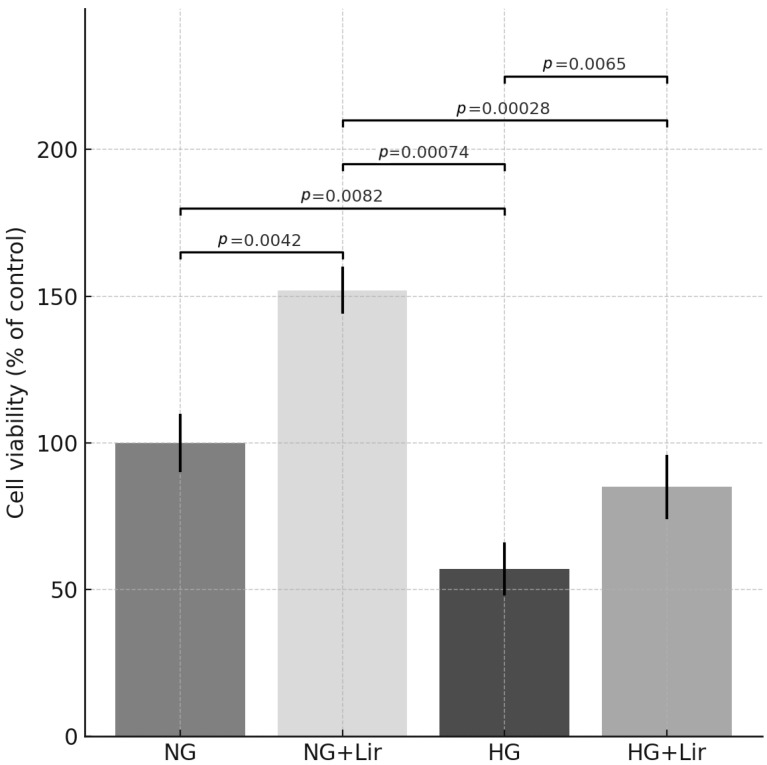
Effect of Lir on cell viability in H9c2 cardiomyocyte cells under hyperglycemic conditions. Data are presented as mean ± SEM. *n* = 6 independent experiments. Statistical analysis was performed using one-way ANOVA followed by Tukey’s post hoc test. Exact *p*-values are indicated in the figure.

The MTT assay was used to measure cell viability to assess the effect on cell viability of Lir. The results showed increased cell viability in the Lir-treated group compared to the control group. The EC_50_ values were calculated as 9.01 ± 0.08 μM for 24 h, 1.07 ± 0.13 μM for 48 h, and 0,81 ± 0.05 μM for 72 h on H9c2 cells (Figure 1 and Figure 2).

### 3.2. TEM Results in H9c2 Cells

**Figure 3:**
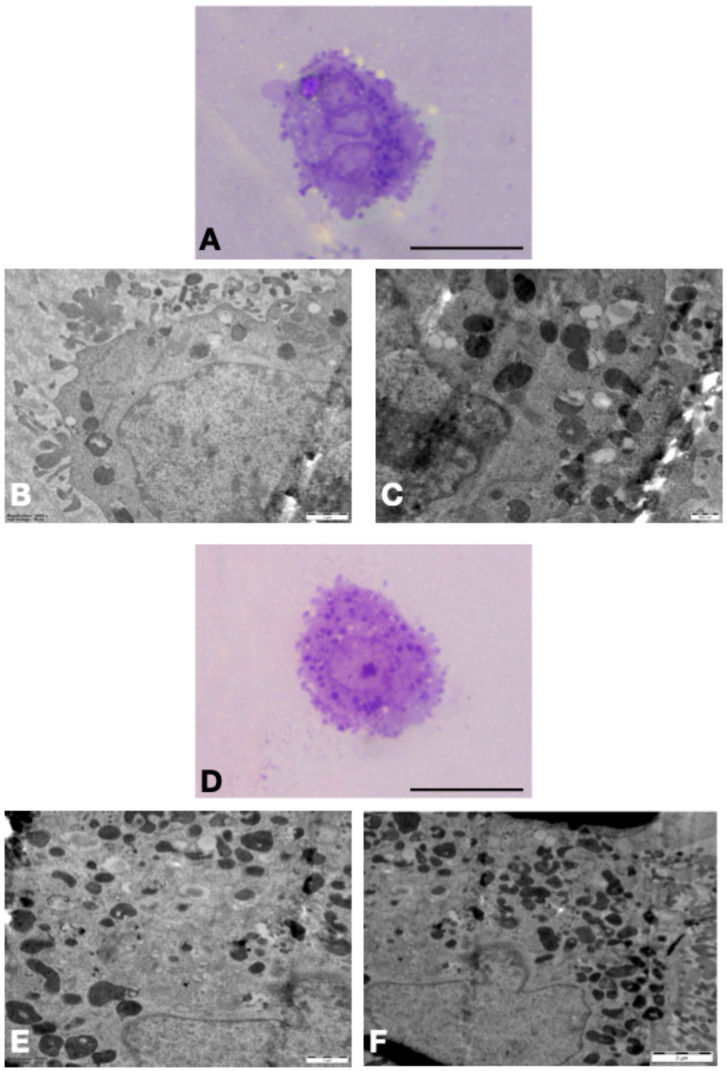
Representative micrographs from semi-thin (**A**,**D**) and thin (**B**,**C**,**E**,**F**) sections of the H9C2 (2-1) cardiomyocyte cell line. (**A**–**C**) Ultrastructural micrographs of the NG + Lir group. (**D**–**F**) Ultrastructural micrographs of the HG + Lir group show a higher number of cytoplasmic granules, vacuolization, and fragmentation. Scale bar: 10 µm for (**A**,**D**), 1 µm for (**B**,**E**), 500 nm for (**C**), 2 µm for (**F**).

Compared to the NG + Lir group, the HG + Lir group exhibited a higher number of cytoplasmic granules. Cells in this group also showed extensive vacuolization and signs of fragmentation. While normal mitochondria were observed in both groups, the HG + Lir group demonstrated notable dilatation of the rough endoplasmic reticulum and a reduced number of pseudopodia (Figure 3).

### 3.3. Effects of Lir on HIF-1α Levels and Oxidative Stress Biomarkers in H9c2 Cells

**Figure 4:**
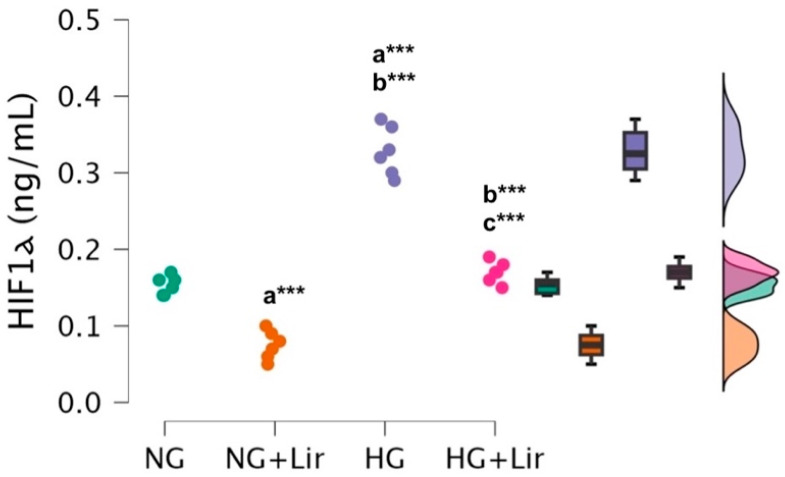
Effects of liraglutide on HIF-1α levels in normoglycemic and hyperglycemic H9c2 cardiomyocyte cells. Individual data points (dots) from independent experiments are shown, together with box plots (median and interquartile range) and violin plots indicating data distribution. (*n* = 6 independent experiments). Exact *p*-values are reported in the text. *** *p* < 0.001.

Lir treatment led to a significant effect on the levels of the HIF-1α (F(3,20) = 157.95, *p* = 4.309 × 10^−14^). Post hoc analysis showed that Lir treatment significantly reduced HIF-1α in both normoglycemic and hyperglycemic conditions. Compared to NG cells (0.153 ± 0.012 ng/mL), NG + Lir cells (0.075 ± 0.019 ng/mL) showed a marked decrease (*p* = 1.218 × 10^−5^). Similarly, HG cells (0.328 ± 0.032 ng/mL) exhibited strongly elevated HIF-1α compared to NG (*p* = 2.196 × 10^−11^), while NG + Lir vs. HG was also highly significant (*p* = 3.841 × 10^−14^). Lir lowered HIF-1α in HG cells (HG + Lir: 0.170 ± 0.014 ng/mL), significantly different from untreated HG (*p* = 1.331 × 10^−10^), and closer to NG levels (*p* = 0.250) (Figure 4).

**Figure 5:**
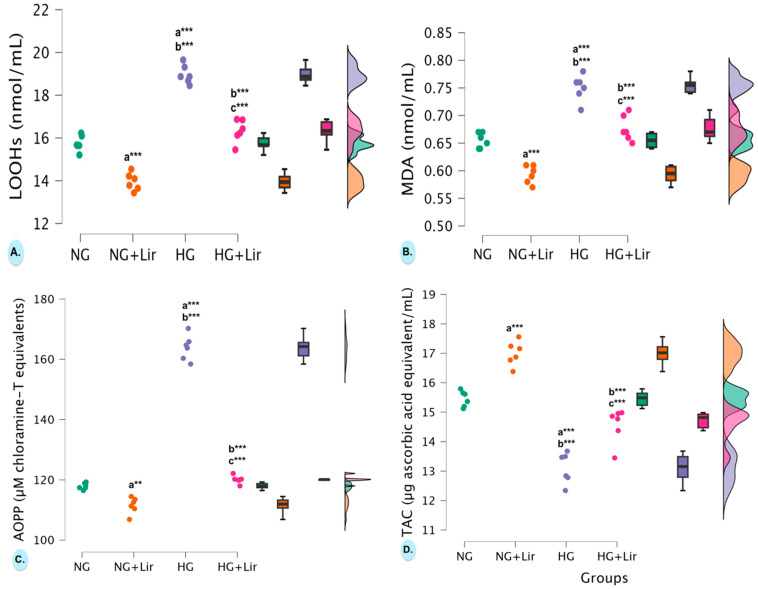
Effects of liraglutide on (**A**) lipid hydroperoxides (LOOHs), (**B**) malondialdehyde (MDA), (**C**) advanced oxidation protein products (AOPP), and (**D**) total antioxidant capacity (TAC) levels in normoglycemic and hyperglycemic H9c2 (2-1) cardiomyocyte cells. Individual data points (dots) from independent experiments are shown, together with box plots (median and interquartile range) and violin plots indicating data distribution. (n = 6 independent experiments). Exact *p*-values are reported in the text. ** *p* < 0.01 and *** *p* < 0.001.

Hyperglycemia significantly increased lipid peroxidation markers. For LOOH, ANOVA yielded F(3,20) = 134.05, *p* = 2.062 × 10^−13^. HG cells (18.971 ± 0.438 nmol/mL) were elevated vs. NG (15.751 ± 0.366 nmol/mL, *p* = 2.862 × 10^−10^). Lir significantly reduced LOOH in both NG (13.953 ± 0.408 nmol/mL, *p* = 4.037 × 10^−6^ vs. NG) and HG cells (16.326 ± 0.528 nmol/mL, *p* = 8.955 × 10^−9^ vs. HG; Figure 5A). For MDA, ANOVA showed F(3,20) = 64.18, *p* = 1.927 × 10^−10^. HG cells (0.750 ± 0.024 nmol/mL) were higher than NG (0.655 ± 0.014 nmol/mL, *p* = 3.573 × 10^−7^). Lir reduced MDA in both NG (0.593 ± 0.016 nmol/mL, *p* = 1.497 × 10^−4^ vs. NG) and HG cells (0.677 ± 0.023 nmol/mL, *p* = 1.607 × 10^−5^ vs. HG). Importantly, post-treatment values in hyperglycemic cells did not significantly differ from untreated normoglycemic controls (*p* = 0.206 for LOOH and *p* = 0.433 for MDA; Figure 5B).

Similarly, AOPP, indicative of protein oxidation, was strongly affected (F(3,20) = 496.00, *p* = 6.192 × 10^−19^). AOPP levels were significantly higher in hyperglycemic cardiomyocytes compared to normoglycemic ones (163.868 ± 4.187 µM chloramine-T equivalents vs. 117.972 ± 1.053 µM chloramine-T equivalents; *p* = 1.921 × 10^−14^). After treatment with Lir, AOPP levels decreased significantly in both glycemic groups (HG + Lir group: 120.083 ± 1.313 µM chloramine-T equivalents and NG + Lir group: 111.547 ± 2.716 µM chloramine-T equivalents) compared to the respective controls (*p* = 1.932 × 10^−14^ vs. HG and *p* = 0.002 vs. NG). In addition, AOPP concentrations in the HG + Lir group were close to those in the NG group (*p* = 1.000) (Figure 5C).

Also, total antioxidant capacity (TAC) was strongly affected (F(3,20) = 73.78, *p* = 5.437 × 10^−11^). TAC was found to be significantly reduced in hyperglycemic cells (13.102 ± 0.526 µg ascorbic acid equivalent/mL) compared to normoglycemic controls (15.453 ± 0.270 µg ascorbic acid equivalent/mL; *p* = 1.602 × 10^−7^). Lir treatment significantly increased TAC levels in both glycemic groups (HG + Lir group: 14.563 ± 0.588 µg ascorbic acid equivalent/mL and NR + Lir: 16.997 ± 0.413 µg ascorbic acid equivalent/mL) compared to their respective controls (HG and NG) (*p* = 1.371 × 10^−4^ vs. HG and *p* = 6.987 × 10^−5^ vs. NG). After Bonferroni correction, TAC levels in cells from the HG + Lir group approached the levels observed in cells from the NG group (*p* = 0.021) (Figure 5D).

## 4. Discussion

The current study demonstrated that Lir significantly enhances cell viability and alleviates oxidative stress in H9c2 cardiomyocytes, particularly under hyperglycemia. The improvement of cell viability, as reflected by the MTT assay and the calculated EC_50_ value of 1.05 ± 0.06 μM, agrees with previous studies that GLP-1R agonists exert cytoprotective effects irrespective of their glucose-lowering effect. Lir was shown to enhance the decrease in H9C2 cell viability and increase cytotoxicity, apoptotic cell percentage, and oxidative stress in H9c2 cells under different experimental conditions [10,14]. These findings build on previous knowledge by demonstrating not only improvement in oxidative stress parameters but also modulation of mitochondrial and hypoxia-related pathways, thereby bridging GLP-1R agonism with fundamental pathways of diabetic cardiomyopathy [19,20]. Our investigation provides additional value over previous studies on H9c2 cells with GLP-1R agonists [10,14] by integrating mitochondrial, oxidative, and hypoxia-related parameters within a single study design.

Accumulating evidence demonstrates that in diabetes, tissues including cardiomyocytes, retina, kidney, pancreatic islets, adipose tissue, skin, and wounds experience hypoxia, which is considered a key contributor to the development and progression of diabetes and its complications [21]. High glucose levels inhibit the stabilization and function of HIF-1 under hypoxic conditions in cardiomyocytes [22]. However, diabetes impairs HIF-1-dependent adaptive hypoxic responses, leading to cellular dysfunction [21]. The mechanisms for this are poorly understood. The attenuation of HIF-1α levels observed here suggests that Lir may also decrease cellular hypoxia or increase oxygen sensing, which is relevant to diabetic cardiomyopathy. Our findings that Lir significantly modulated HIF-1α levels under both normoglycemic and hyperglycemic conditions suggest that the drug may influence cellular responses associated with hypoxic stress, potentially contributing to improved cellular resilience in the context of diabetes. Fatty acids have also recently been reported to play another role in preventing the inhibition of HIF-1 in cardiomyocytes with a T2DM-like phenotype [23]. Specifically, pharmacologic reversal of HIF-1 inhibition through the inhibition of prolyl hydroxylases (PHDs) in diabetic hearts was shown to improve cardiac recovery following an ischemic event in a model of type 2 diabetes in rats [23]. These findings also validate the potential of HIF-1 recovery in the diabetic heart and, importantly, point to Lir as an excellent modulator of this pathway. Significantly, the pharmacological activity of Lir upon the pancreas islets has already been attributed to its angiogenic activity, with which HIF-1-mediated actions have intimate relationships. The ability of glucagon-like peptide-1 analogues to improve revascularization of transplanted islets, primarily via HIF-1–dependent mechanisms, highlights their potential in preserving graft function and survival. This also raises the possibility that targeting HIF-1 pathways may represent a broader strategy for mitigating hypoxia-associated complications in diabetes [24]. This indicates that Lir’s effects on HIF-1α likely reflect a general modulation of cellular stress responses, rather than a cardiomyocyte-specific mechanism, potentially contributing to improved cellular adaptation under hypoxia-related conditions.

In concurrence with the proven effect of hyperglycemia on oxidative stress, our study showed that LOOH and MDA, as lipid peroxidation markers, were significantly greater in hyperglycemic cardiomyocytes compared to normoglycemic controls. This aligns with previous findings indicating that chronic hyperglycemia promotes excessive ROS production and lipid membrane damage [25,26]. In the current study, importantly, Lir treatment significantly reduced both LOOH and MDA levels in hyperglycemic and normoglycemic conditions, suggesting a strong antioxidant effect. Interestingly, Lir, in addition to reversing hyperglycemic cell markers of oxidative damage, also reversed these to the control range of normoglycemia. These results are consistent with earlier reports that Lir inhibits oxidative damage through improving mitochondrial function and strengthening endogenous antioxidant defenses [15,27,28]. Lir’s capacity to normalize lipid peroxide markers also indicates its capacity to detoxify cell damage under hyperglycemia-induced diabetic cardiomyopathy. These results are consistent with the findings of Zhang et al. [15], which demonstrated that Lir inhibited high glucose-induced oxidative damage and apoptosis in neonatal rat cardiomyocytes dramatically. In the current research, Lir reduced the synthesis of ROS, increased mitochondrial membrane potential, and inhibited pro-apoptotic signaling pathways. These are consistent with our observations of inhibited lipid peroxidation (LOOH and MDA), increased total antioxidant activity, as well as maintenance of cellular ultrastructure after Lir administration under the condition of hyperglycemia. These findings are all consistent with the view that Lir exerts direct cardioprotective effects by countering oxidative stress and inhibiting hyperglycemia-induced tissue damage at both molecular and ultrastructural levels.

DM is associated with the overproduction of advanced oxidation protein products (AOPP). AOPPs are stable products of protein oxidative damage and accumulate in hyperglycemic conditions, fostering cellular dysfunction and inflammation [5]. In the current study, hyperglycemia significantly raised AOPP levels, while Lir treatment restored the levels close to those of normoglycemic controls, suggesting a powerful protective effect. Our findings showed that Lir significantly reduced AOPP levels, indicating less protein oxidation in both normoglycemic and hyperglycemic cardiomyocytes. Interestingly, Tonon Firmino et al. [29] established that low-dose Lir reduced AOPP levels in the renal tissue of hypertensive female rats but that high-dose Lir could not have this antioxidant activity and was associated with early renal damage markers. This contrast supports the idea that Lir’s antioxidant potential, particularly in reducing protein oxidation, may be dose-dependent and tissue-specific. These observations highlight the importance of evaluating optimal dosing to maximize protective effects while minimizing potential tissue-specific risks. Our findings are supported by Gaballah et al. [30], who discovered that Lir alone and in combination with quercetin lowered AOPP levels substantially in a rat model of T2DM induced by streptozotocin and high-fat diet. Their study highlights Lir’s capacity to reverse protein oxidative damage systemically in diabetic conditions, again affirming our findings in cardiac cells. Together, all these findings suggest that Lir’s antioxidative action is preserved across different tissues and across different models of diabetes, in particular, in its ability to inhibit protein oxidation. These results lend further credence to the view that Lir mitigates oxidative stress via not just the inhibition of lipid peroxidation but also by its preservation of protein integrity, pivotal to maintaining cell homeostasis. That Lir can normalize AOPP levels is possibly a reflection of its ability to suppress ROS generation and augment antioxidant defense in cardiac cells exposed to diabetic stressors.

Our findings demonstrated that hyperglycemia markedly reduced TAC, reflecting impaired cellular resistance to oxidative stress. Notably, liraglutide treatment substantially increased TAC in both hyperglycemic and normoglycemic cardiomyocytes, with levels in the HG + Lir group nearly restored to those observed under normoglycemic conditions. These results suggest that liraglutide not only prevents oxidative damage but also contributes to the re-establishment of antioxidant homeostasis within cardiomyocytes. Ding et al. [31] demonstrated Lir inhibited vessel restenosis in diabetic rabbits partially through inhibition of oxidation damage through modulation of the TGF-β/Smad3 pathway. Their study demonstrated that Lir inhibited vascular tissue inflammation and oxidation, resulting in increased vascular healing. This is aligned with our discovery that Lir increased TAC and blocked lipid and protein oxidation in cardiomyocytes, suggesting Lir’s antioxidant activity is tissue-protecting and mechanism-related to many of the diabetic complication syndromes. Additional evidence of Lir’s protective antioxidant activity, that of Kuo et al. [14] demonstrated Lir decreased lipid droplet accumulation and myocardial fibrosis of diabetic cardiomyopathy partially due to prevention of oxidation damage and improvement of mitochondrial function. Their study proves Lir’s control of oxidation damage at structural and metabolic levels in cardiac tissue. In the present study, TEM micrographs revealed partial maintenance of cellular architecture in the HG + Lir group with higher granules, maintained mitochondrial morphology, and dilatation of rough endoplasmic reticulum. Mitochondrial dysfunction is increasingly recognized as a central feature of diabetes-associated cardiac injury, driving impaired ATP synthesis, increased ROS generation, and disturbed calcium handling. In the current investigation, Lir also preserved mitochondrial membrane potential, reduced ROS production, and preserved organelle ultrastructure. These effects are consistent with data showing that GLP-1R agonists exert mitochondrial protective actions by regulating bioenergetics and quality control processes [32,33,34]. This suggests that Lir protection is not confined to glycemic management but includes direct mitochondrial stabilization.

## 5. Conclusions

These results recognize liraglutide as a promising dual-action therapeutic candidate for diabetic cardiomyopathy through glucose-independent cryoprotection and regulation of mitochondrial and hypoxia pathways. Limitations are to be mentioned, however. First, the use of the H9c2 in vitro model prevents direct extrapolation to human cardiomyocytes. Second, we did not assess long-term or in vivo effects, which are necessary for establishing translational potential. Third, a limitation of the present study is the absence of osmotic controls (e.g., mannitol) in high-glucose experiments and vehicle controls for liraglutide. Fourth, while mitochondrial and hypoxia-dependent mechanisms were implicated, further studies with direct assessments of mitophagy, respiratory chain activity, and calcium flux regulation are required to elucidate the exact molecular pathways. Finally, although our mechanistic claim involves GLP-1 receptor-mediated protection, we were not able to directly demonstrate GLP-1R expression in H9c2 cells due to budgetary constraints. Future investigations incorporating receptor expression analyses are needed to validate this mechanism.

In summary, this research provides novel evidence that liraglutide exerts cardioprotective effects through reducing oxidative damage, preserving mitochondrial function, and modulating hypoxia signaling in cardiomyocytes exposed to hyperglycemia. These findings provide a new perspective on the role of GLP-1R agonists in diabetic cardiomyopathy and highlight their therapeutic potential beyond glucose regulation.

## Data Availability

The datasets generated and/or analyzed in the current study are available from the corresponding author upon reasonable request. All data supporting the findings of this study are included within the article.

## Abbreviations

The following abbreviations are used in this manuscript:

AOPP	Advanced oxidation protein products
DM	Diabetes mellitus
GLP-1	Glucagon-like peptide-1
GLP-1R	Glucagon-like peptide-1 receptor
HG	Hyperglycemia
HIF-1α	Hypoxia-inducible factor-1α
Lir	Liraglutide
LOOH	Lipid hydroperoxides
MDA	Malondialdehyde
NG	Normoglycemia
ROS	Reactive oxygen species
SGLT2	Sodium-glucose cotransporter 2
TAC	Total antioxidant capacity
T2DM	Type 2 diabetes mellitus
